# Single-Atom Catalysts for Biotherapy Applications: A Systematic Review

**DOI:** 10.3390/nano10122518

**Published:** 2020-12-15

**Authors:** Shan Jiang, Chengfei Zhang, Ting Zou

**Affiliations:** 1 School of Stomatology, Shenzhen University Health Science Center, Shenzhen 518060, China; jshan@szu.edu.cn; 2 Restorative Dental Science, Faculty of Dentistry, The University of Hong Kong, Pokfulam, Hong Kong, China; zhangcf@hku.hk

**Keywords:** single-atom catalysts, enzyme-like, reactive oxide species, biotherapy, disease

## Abstract

Single-atom catalysts (SACs), as atomically dispersed metal active sites anchored or coordinated on suitable supports, demonstrate large potential for use in therapeutic applications. SACs have structural features similar to those of natural enzyme, while exhibiting remarkable catalytic activity, desirable stability, and excellent selectivity. This systematic review aims to synthesize evidence on SACs’ biotherapy applications. Three databases (PubMed/MEDLINE, ISI Web of Science, and ScienceDirect) were searched to identify the studies that investigated the therapeutic efficacy of SACs. A total of 12 studies that fulfilled the inclusion criteria were included and reviewed, and the key findings were qualitatively synthesized. Overall, various SACs were investigated for biotherapy applications, including anticancer, anti-infection (antibacterial), and anti-inflammatory applications; brain trauma therapies, and oxidative-stress cytoprotection applications. All of the included studies showed that the synthesized SACs demonstrated superior therapeutic effects compared with their respective controls. Among the 12 studies reviewed, 11 studies showed satisfied biocompatibility of the applied SACs, whereas minimal cytotoxicity was reported in 1 study. Collectively, the reviewed studies indicated that SACs exhibited considerable promise in the field of biotherapy. Additional studies are needed for a better understanding of the effect of SACs in the treatment of various diseases.

## 1. Introduction

Single-atom catalysts (SACs), a novel class of catalysts in which all of the isolated, catalytically active metal atoms are stabilized by a support, were first proposed by Zhang and coworkers in 2011 [1]. Subsequently, the state-of-the-art SACs have been proven to exhibit superb performance in various chemical reactions on the basis of the optimized use of metal atoms with well-defined active centers [2,3,4]. Taking advantage of their properties, including but not limited to their extraordinary catalytic activity, outstanding selectivity, desirable stability, and 100% atom utilization, SACs offer the merits of both heterogeneous and homogeneous catalysts, and thus have attracted extensive attention in regard to chemical catalysis in the petrochemical industry, environmental protection, energy conversion, chemical transformation, and biomedical applications [5,6,7,8,9]. A considerable amount of evidence has demonstrated that SACs are powerful and effective in many typical heterogeneous catalytic reactions, such as electrochemical reactions, water-gas shift reactions, and hydrogenation reactions [10,11,12]. Moreover, SACs are low cost, abundant, and environmentally friendly resources [13,14].

In 2018, SACs were introduced into the field of biotherapy [15]. Despite the sizeable amount of evidence in industrial, energy, and ecological applications, the potential of SACs in biotherapy is much less understood. To the best of the authors’ knowledge, there is no systematic review on SACs in biotherapy applications that has been published. In a mini review published by Jiao and coworkers in 2020 [16], they summarized the recent progress in the synthesis, characterization, and application of SACs, which showed distinct advantages in environmental protection and biomedical applications. Nevertheless, they did not systematically analyze the therapeutic efficacy of SACs in biotherapy applications. In addition, it may have only captured a part of the available evidence without a systematic search of relevant databases. 

In recent years, SACs have been synthesized for several biotherapy applications in a growing number of studies, such as antitumor therapies, anti-infection applications, and brain trauma therapies. Therefore, to facilitate a better understanding of the potential applications of SACs in biotherapy, this article has systematically reviewed the available literature to summarize the current evidence on the effect of SACs in biotherapy applications.

## 2. Materials and Methods 

### 2.1. Search Strategy

Three scientific databases (PubMed/MEDLINE, ISI Web of Science, and ScienceDirect) were searched for papers published between September 2011 (the year when SACs were developed) and October 2020. Only studies published in English were considered for inclusion. The following search keywords were used: (((single atom catalyst) or (single atom) or (atomically dispersed) or (single-atom) or (single-atom catalyst) or (single-atom catalysis) or (single atom catalysis) or (single-site heterogeneous catalyst)) AND (biotherapy OR therapy OR infection OR infectious disease OR disinfection OR antibacterial OR antibacterial activity OR antimicrobial activity OR antimicrobial OR microbial OR biofilm OR bacterial OR biological activity OR tumor OR antitumor OR cancer OR anticancer OR disease OR trauma)).

### 2.2. Eligibility Criteria 

The study was included if it simultaneously met the following criteria: (1) the study should report the characterization of the synthesized SACs, which measured the structure of the SACs and confirmed the existence of single-metal atoms; (2) the study should refer to biotherapy applications of the SACs; and (3) the study should be peer-reviewed original research publications. A study was excluded if the atomically dispersed catalysts were in the form of atomic clusters. In addition, if the study only mentioned the potential therapeutic application of SACs but without in vivo and/or in vitro bioassays, it was excluded. Third, editorials, opinion articles, conference abstracts, scientific statements, protocols, guidelines, or review articles, or non-English publications were also excluded.

### 2.3. Study Selection and Data Extraction

All retrieved papers were first screened on the basis of their titles and abstracts. Those that were clearly ineligible were excluded. Potentially eligible full-text articles were obtained and assessed for eligibility. Additionally, reference tracking was carried out in the full text of these papers. A data extraction was prepared pro forma to summarize the key information of each included study (source, SAC type, support material, active site, synthesis method, characterization techniques, biocompatibility/biosafety, application model, in vitro/in vivo experiment, activity, mechanism, and main findings). 

## 3. Results

### 3.1. Systematic Search Outcomes

The search of the three databases and the bibliographies of papers yielded 900 papers after excluding duplicate papers. Through screening by titles and abstracts, 25 studies were potentially included. Finally, 12 studies were included in this systematic review. The inter-reviewer reliability was high for both the title/abstract screening and full-text assessment processes (kappa =0.84 and kappa =0.87, respectively).

### 3.2. Characteristics of the Chosen Studies

The key characteristics of the included literature are detailed in Table 1, Table 2 and Table 3. The included studies were published in 2019 and 2020. The most common biotherapy applications were as an anticancer treatment (five studies), followed by an anti-infection therapy (four studies), an oxidative stress cytoprotection application (two studies), and a brain trauma therapy (one study). 

### 3.3. Fabrication and Characterization of the SAC

The fabrication techniques and key characterization in the included studies have been used to synthesize and confirm the SACs and are summarized in Table 1. “Isolation-pyrolysis” is the most widely used path to fabricate SACs to disperse metal atoms on a 2D support. For instance, FeN_5_ single-atom nanozymes with carbon nanoframe-confined (SA/CNF), zinc-based single atom nanozyme (PMCS SA), single iron atoms anchored in nitrogen-doped amorphous carbon (SAF NCs), Co/PMCS, PEGylated single-atom Fe-containing nanocatalysts (PSAF NCs) Fe-N/C SACs, Fe-SAs/NC were developed through the use of pyrolysis [15,17,18,19,20,21,22]. The deposition/coprecipitation strategy is also a common approach for SAC preparation, which was used to prepare carbon dot-supported atomically dispersed gold (MitoCAT-g), porphyrin-like single-atom Fe(III) metal-organic framework (P-MOF), and Pt/CeO_2_ [23,24,25]. Apart from these two methods, SACs can be prepared via self-assembly, exemplified by OxgeMCC-r SAE, and multistep reactions, exemplified by N-doped carbon sphere doped with a single-atom copper species (Cu-HNCS) [26,27]. 

The type of SACs included single-atom nanozymes with carbon nanoframe-confined FeN_5_, single iron atom catalyst (Fe SAEs), zinc-based single-atom nanozyme (PMCS), single iron atoms anchored in nitrogen-doped amorphous carbon (SAF NCs), atomically dispersed Fe-N_4_ immobilized on a carbon substrate (Fe-N/C SACs), nitrogen-doped carbon-supported atomically dispersed Co-porphyrin centers (Co/PMCS), PEGylated single-atom Fe-containing nanocatalysts (PSAF NCs), carbon-dot-supported gold (Au/CDs), porphyrin-like single-atom Fe(III), hollow N-doped carbon sphere doped with a single-atom copper species (Cu-HNCS), single-atom Ru using Mn_3_[Co(CN)_6_]_2_ metal-organic framework (MOF) as the support material, and single-atom Pt/CeO_2_.

Aberration-corrected high-angle annular dark-field scanning transmission electron microscopy (HAADF-STEM) and scanning tunneling microscopy (STM) are the most commonly used characterization techniques to assess SACs because these techniques can directly observe the single-atom metals on supports. The detailed structural information (coordination environment, binding mode, and oxidation states) of single-metal sites in SACs was further investigated by synchrotron radiation-based X-ray absorption near-edge structure (XANES) and extended X-ray absorption fine structure (EXAFS) spectra.

**Figure 1:**
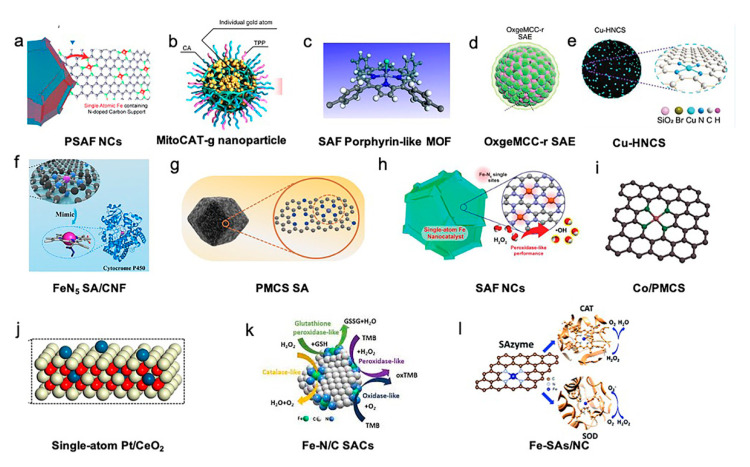
Structures of single-atom catalysts (SACs) in biotherapy applications. (**a**) PEGylated single-atom Fe-containing nanocatalysts (PSAF NCs). Adapted with permission from [21]. Copyright 2019, American Chemical Society. (**b**) Carbon-dot-supported atomically dispersed gold (MitoCAT-g). Adapted with permission from [23]. Copyright 2019, Nature Publishing Group. (**c**) Crystal structure of an Fe porphyrin center in P-MOF. Adapted with permission from [24]. Copyright 2019, American Chemical Society. (**d**) Catalytically active single-atom Ru sites anchored in Mn_3_[Co(CN)_6_]_2_ loading with Ce6 (MCC) with an outer PVP protection layer (OxgeMCC-r). Adapted with permission from [26]. Copyright 2020, Nature Publishing Group. (**e**) Structure of hollow nitrogen-doped carbon sphere doped with a single-atom copper species (Cu-HNCS). Adapted with permission from [27]. Copyright 2020, Wiley-VCH. (**f**) Carbon nanoframe-confined atomically dispersed Fe sites with axial five-N coordination to mimic the active center of cytochrome P450. Adapted with permission from [17]. Copyright 2019, American Association for the Advancement of Science. (**g**) Porphyrin-like structural model of zinc-based single-atom nanozyme (PMCS). Adapted with permission from [18]. Copyright 2019, Wiley-VCH. (**h**) Single-atom Fe nanocatalysts (SAF NCs). Adapted with permission from [18]. Copyright 2019, Wiley-VCH. (**i**) Model of Co/PMCS. Co (purple), N (green), and C (gray). Adapted with permission from [20]. Copyright 2019, Wiley-VCH. (**j**) Single-atom Pt/CeO_2_ nanozymes. Adapted with permission from [25]. Copyright 2019, American Chemical Society. (**k**) Schematic diagram of the intrinsic activity of Fe-N/C SACs mimicking peroxidase, oxidase, catalase, and glutathione peroxidase enzymes. Adapted with permission from [22]. Copyright 2019, The Royal Society of Chemistry. (**l**) Atomically dispersed Fe-N_4_ sites anchored on N-doped porous carbon materials (Fe-SAs/NC), which mimic two antioxidative enzymes: catalase (CAT) and superoxide dismutase (SOD). Adapted with permission from [15]. Copyright 2018, The Royal Society of Chemistry.

The active metal atoms of SACs included Fe, Zn, Co, Cu, Pt, Au, and Ru [15,17,18,19,20,22,23,24,25,26,27]. The selected support materials of the SACs included carbon materials, such as carbon nanoframes, carbon nanospheres, nitrogen-doped carbon, hollow nitrogen-doped carbon spheres, carbon dots, MOFs, Mn_3_[Co(CN_6_)_6_]_2_, and CeO_2_ [15,17,18,19,20,21,23,24,25,26,27]. The structures of the included SACs are presented in Figure 1. 

### 3.4. Biocompatibility of the SACs

The biocompatibility/biosafety of the synthesized SACs is one of the critical factors for their biotherapy application; thus, the biocompatibility of SACs was reported in all of the included studies. In general, the vast majority of the studies (11 out of 12) reported the favorable biocompatibility of SACs. Minimal cytotoxicity was reported in 1 remaining study. The cytocompatibility studies included a CCK8 assay [17,18,23,27] and/or Live/Dead assay [24] or an MTT assay [15,20,22,25] or a Cell Titer-Fluor assay [26], while studies conducted by Huo and coworkers [19,21] revealed the biocompatibilities of SAF NCs via the hematoxylin and eosin (HE) staining of major organs dissected from mice in different therapeutic groups. In regard to cytocompatibility, the single-atom catalyst of SAs/NC with bifunctional antioxidative enzymes for oxidative-stress cytoprotection exhibited the minimal cytotoxicity, which was reported by Ma and coworkers [15]. 

### 3.5. Application of SACs in Cancer Treatments 

**Figure 2:**
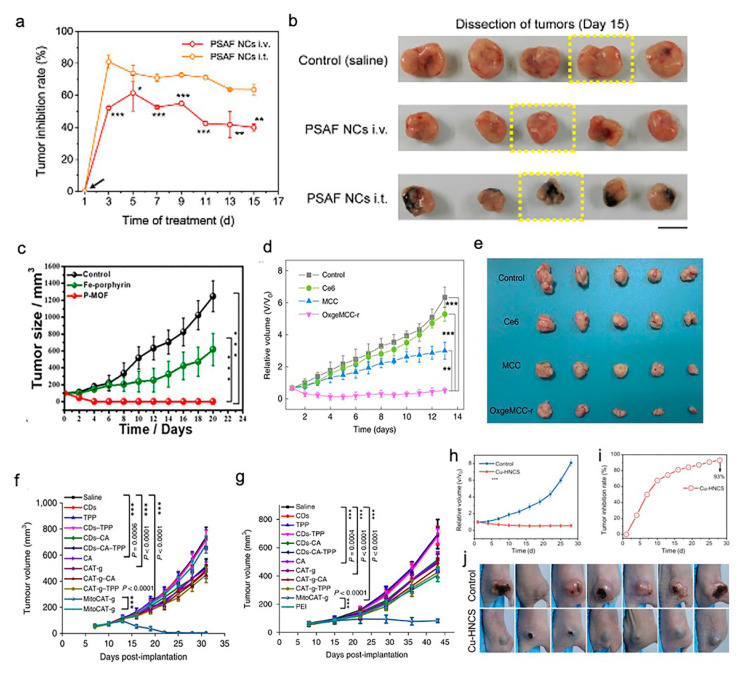
Effect of SACs in cancer treatments. (**a**) Relative tumor inhibition rates of tumors treated with PEGylated single-atom Fe-containing nanocatalysts (PSAF NCs) intravenously and intratumorally, in contrast to the control group, (**b**) digital photographs of the dissected tumors of the control, PSAF NCs i.v. and PSAF NCs i.t. groups. (**a**,**b**) Adapted with permission from [21]. Copyright 2019, American Chemical Society. (**c**) Tumor growth curves with different treatments. Adapted with permission from [24]. Copyright 2019, American Chemical Society. (**d**) Relative tumor volumes in mice after various treatments (control, Ce6, MCC, and OxgeMCC-r), (**e**) Photographic images of tumors excised from different groups after various treatments indicated. (**d**,**e**) Adapted with permission from [26]. Copyright 2020, Nature Publishing Group. (**f**) Tumor growth in the MitoCAT-g-treated group compared with the saline control group in a subcutaneous tumor model. (**g**) Tumor volumes in the different treatment groups in the orthotopic hepatic patient-derived xenograft (PDX) tumor model in non-obese diabetic/severe combined immune-deficiency (NOD-SCID) mice. (**f**) and (**g**) Adapted with permission from [23]. Copyright 2019, Nature Publishing Group. (**h**) Tumor proliferation curves using parallel catalytic therapy in a subcutaneous tumor model. (**i**) Relative tumor inhibition rates. (**j**) Photographs of 4T1 tumors 28 days after treating bagg albino, laboratory-bred strain of the house mouse/c (BALB/c) nude mice with blank hydrogels or Cu-HNCS hydrogels. (**h**–**j**) Adapted with permission from [27]. Copyright 2020, Wiley-VCH.

SACs were applied as a cancer therapy in five studies (Table 1) [21,23,24,26,27]. These studies targeted breast cancer (four studies) and liver cancer (one study). HeLa cells (two studies), 4T1 tumor cells (three studies), HEK 293 cells (one study), and HepG-2 cells (one study) were used in their in vitro assays. For studies targeting breast cancer, 4T1 tumor-bearing mice (three studies) and HeLa-bearing tumor mice (one study) were used. In addition, a mouse model based on xenografted HepG-2 tumors was used in a study focused on liver cancer. In general, all of the five studies reported that the synthesized SACs, which exhibited desirable biosafety, were highly efficient in both the in vitro and in vivo cancer treatments. PEGylated single-atom Fe-containing nanocatalysts (PSAF NCs), a metal-organic framework (MOF) rich in porphyrin-like single-atom Fe(III) (P-MOF), and single-atom Ru supported by a Mn_3_[Co(CN)_6_]_2_ MOF (OxgeMCC-r SAE) outperformed the saline or phosphate-Buffered Saline (PBS) control in inhibiting the growth profiles of breast tumors (Figure 2a–e) [21,24,26]. It is worth mentioning that both OxgeMCC-r SAE and P-MOF enhanced the therapeutic outcome of photodynamic therapy (PDT) in breast cancer via two different mechanisms. The OxgeMCC-r SAE exhibited a high loading capacity of Ce6 photosensitizer. On the other hand, the spin state of Fe(III) in P-MOF under NIR would facilitate the generation of singlet oxygen, which promoted PDT. Moreover, they also reported that P-MOF could serve as an agent for a photothermal therapy (PTT) cancer treatment, as well as photoacoustic imaging (PAI) of breast tumors. In addition, Gong et al. found that carbon dot-supported atomically dispersed gold (MitoCAT-g) significantly suppressed tumor growth in subcutaneous and orthotopic patient-derived xenograft hepatocellular carcinoma models without adverse effects or toxicity, when compared with the saline contro1 (Figure 2f,g) [23]. Recently, a study conducted by Lu and coworkers reported that the bioinspired Cu-HNCS could generate two types of reactive oxygen species (ROS) (O_2_•^−^ and •OH) through two parallel reactions, which significantly enhanced the efficacy of the parallel catalytic of tumors in in vitro and in vivo (Figure 2h–j) [27]. 

### 3.6. Application of SACs in Anti-Infection Therapies

**Figure 3:**
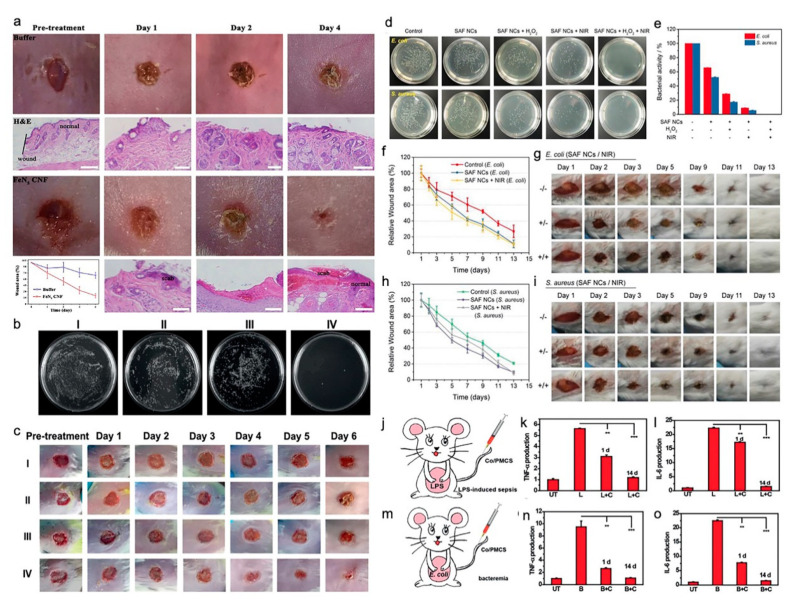
Effect of SACs in anti-infection therapies. (**a**) Photographs of the in vivo mice wound model. Photographs of the *Escherichia coli* infected wound treated with PBS buffer and carbon nanoframe-confined FeN_5_ (FeN_5_ CNF) solutions at the pre-treatment stage and after 1–3 days, and their corresponding histologic analyses. Related wound size of mice in each group after different treatments (diagram in the left corner). Adapted with permission from [17]. Copyright 2019, American Association for the Advancement of Science. (**b**) Photographs of bacterial colonies formed by *Pseudomonas aeruginosa* after exposure to (I) NaAc buffer; (II) NaAc buffer + H_2_O_2_; (III) PMCS, and (IV) PMCS + H_2_O_2_. (**c**) Photographs of *P. aeruginosa*-infected wound treated with (I)–(IV) after different lengths of time. (**b**,**c**) Adapted with permission from [18]. Copyright 2019, Wiley-VCH. (**d**) Digital photographs of remaining bacteria-inoculated agar plates. (**e**) Bacterial activity of treatment. (**f**) Relative wound area. (**g**) Digital photographs of mice in *E. coli*-infected group. (**h**) Relative wound area. (**i**) Digital photographs of mice in *Staphylococcus aureus*-infected group. (**d**–**i**) Adapted with permission from [19]. Copyright 2019, Wiley-VCH. (**j**) Illustration of in vivo LPS-induced sepsis model. (**k**–**l**) Detection of TNF-α and IL-6 in vivo in different groups (UT: untreated, L: LPS, C: Co/PMCS). (**m**) Illustration of in vivo bacteremia model, (**n**,**o**) Detection of TNF-α and IL-6 in vivo in different groups (UT: untreated, B: bacteremia, C: Co/PMCS). (**j**–**o**) Adapted with permission from [20]. Copyright 2019, Wiley-VCH.

There were four studies on the application of SACs for anti-infection therapies (Table 2) [17,18,19,20]. Among them, three studies targeted wound disinfection applications [17,18,19], and one study examined sepsis management [20]. These four studies consistently showed that the synthesized SACs demonstrated superior antibacterial activity without, or at least very little, toxicity. In comparison to the control group, single-atom nanozymes with carbon nanoframe-confined FeN_5_ (FeN_5_ SA/CNF), zinc-based single atom nanozyme (PMCS SAzyme), and single iron atoms anchored in nitrogen-doped carbon (SAF NCs) exhibited very high statistically significant differences in inhibiting the growth of *Pseudomonas aeruginosa (P. aeruginosa*), *Escherichia coli* (*E. coli*)/*Staphylococcus aureus* (*S. aureus*) in vitro, and significantly promoted wound healing without toxicity in vivo. Cao and coworkers reported that the synthesized Co/PMCS could significantly reduce the number of bacteria in the liver, lung, kidney, intestines, and blood of *E. coli-*induced bacteremia in mice, as well as reducing the white blood cells (WBC), ROS, alanine transaminase (ALT), RNS, IL-6, TNF-α, Lym, Neu, and urea, in organs and blood, when compared with those of the PBS-treated control group. Additionally, they found that all the blood indexes of lipopolysaccharide (LPS)-induced sepsis mice in the Co/PMCS-treated group were reduced to normal. In other words, Co/PMCS could significantly eliminate the systematic production of reactive oxygen and nitrogen species (RONS) and lower proinflammatory cytokine levels compared with those of the PBS-treated group, thereby indicating that Co/PMCS could promote the recovery of multiple organ functions in sepsis mice (Figure 3) [20].

### 3.7. Other Applications of SACs 

**Figure 4:**
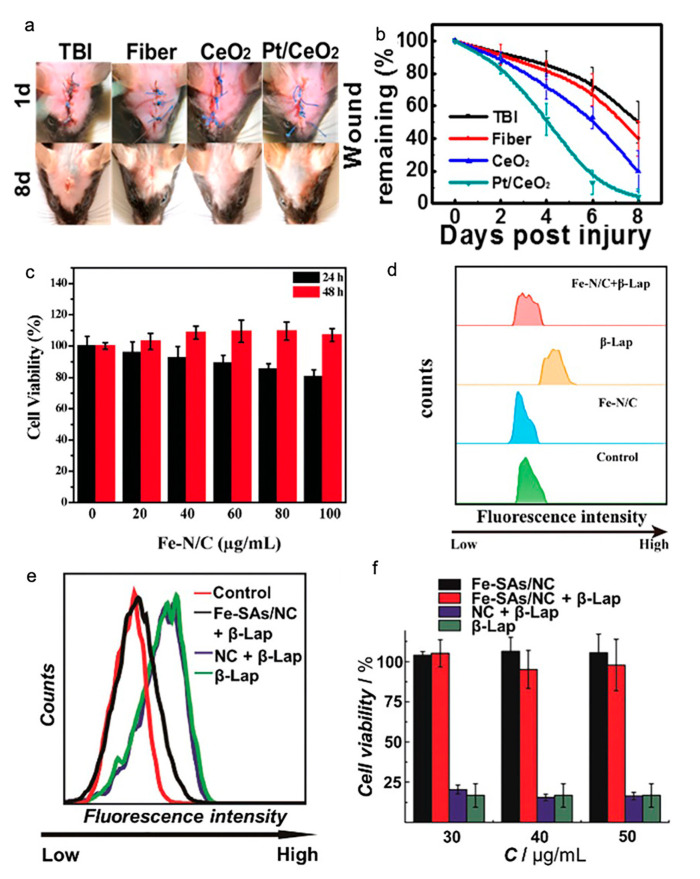
Effect of SACs in brain trauma and cytoprotection applications. (**a**) Representative photos of wound healing. (**b**) Residual wound over time with and without the treatment of a nanozyme-based bandage. (**a**,**b**) Adapted with permission from [25]. Copyright 2019, American Chemical Society. (**c**) HeLa cell viability at different concentrations of Fe-N/C SACs, (**d**) Scavenging effect of Fe-N/C SACs (100 μg mL^−1^) on ROS in β-Lap treated HeLa cells, as detected by flow cytometry. (**c**,**d**) Adapted with permission from [22]. Copyright 2019, The Royal Society of Chemistry. (**e**) Intracellular reactive oxygen species (ROS) scavenging by 30 µg mL^−1^ NC (purple line) and Fe-SAs/NC (black line) in β-Lap-treated cancer cells, as detected by a DCFDA-H_2_ assay. (**f**) Effects of different concentrations of NC (purple line) and Fe-SAs/NC (black line) on the viability of β-Lap-treated cancer cells. (**e**,**f**) Adapted with permission from [15]. Copyright Royal Society of Chemistry.

SACs were also applied for the noninvasive treatment of neurotrauma (Table 3). Yan and coworkers synthesized single-atom Pt/CeO_2_ as a therapeutic agent with good biocompatibility to treat brain trauma. It was reported that Pt/CeO_2_ could significantly decrease inflammatory factors such as interleukin (IL)-1β, IL-6, and tumor necrosis factor (TNF)-α in vitro. Moreover, it could significantly reduce the wound size and area and the expression of inflammatory cytokines and excess peroxidation, while recovering the superoxide dismutase SOD) and MMP-9 levels and activating astrocytes and microglia; these actions lead to a decrease in the overall neuroinflammation in traumatic brain injury (TBI) mice compared with that in the untreated group (Figure 4) [25]. 

Two other studies reported that SACs could efficiently protect HeLa cells from damage by cellular oxidative stress by scavenging intracellular ROS, thereby providing an opportunity for an ROS-related disease treatment (Table 3) [15,22]. It was indicated that both the synthesized single atom catalysts (Fe-N/C SACs) and N-doped porous carbon (Fe-SAs/NC) exhibited a higher ability to remove excess ROS that were generated from cells under oxidative stress compared with that of the control group (Figure 4). 

**Table 1:** Application of SACs on cancer treatment.

Author	Type of SAC	Support Material	Active Site (Single Atom)	Synthesis Method	Characterization Techniques	Biocompatibility BIOSAFETY	Type of Cancer	Targeted Cells	In Vitro/ In Vivo	Activity	Mechanism	Main Findings
Huo et al. (2019) [21]	PEGylated single-atom Fe-containing nanocatalysts (PSAF NCs)	N-doped carbon	Fe (1.54 wt.% loading ratio)	High-temperature pyrolysis	XRD, Aberration-corrected high-angle annular dark-field scanning transmission electron microscopy (HAADF-STEM), EDS, Inductively coupled plasma - optical emission spectrometry (ICP-OES), X-ray absorption spectroscopy (XAS), X-ray absorption near-edge structure (XANES), Extended X-ray absorption fine structure (EXAFS),	Satisfied (body weight/organ image)	Breast cancer (4T1 tumor -bearing mice)	4T1 tumor cell	Both	SAF NCs produced high overall •OH (27.75–54.21%	Fenton reaction to generate abundant toxic •OH	PSAF NCs could lead to the apoptotic cell death of malignant tumors, induce accumulation of lipid peroxides, tumor cell ferroptosis, which result in a remarkable tumor impression.
Gong et al. (2019) [23]	Carbon-dot supported gold (Au/CDs)	Carbon-dot	Au (15.3 wt%)	Deposition/precipitation	TEM, HAADF, XANES, EXAFS	Good blood compatibility	Liver cancer (HepG-2 tumor-bearing mice)	HepG-2 cancer cell	Both	MitoCAT-g causes glutathione (GSH) depletion first, followed by ROS production	Generation of reactive oxygen species (ROS) (MitoCAT-g is the amplification of oxidative stress)	Carbon-dot-supported atomically dispersed gold as a mitochondrial oxidative stress amplifier inhibits tumor growth in subcutaneous and orthotopic patient-derived xenograft hepatocellular carcinoma models.
Wang et al. (2019) [24]	porphyrin-like single atom Fe (III)	MOF	Fe (1.22 wt%)	Deposition/precipitation	XRD, TEM, XAFS, XANES, EXAFS,	Excellent biocompatibility toward Hela cells	Breast cancer (Hela-bearing mice)	Hela cell and MCF-7 cell	Both	Good catalytic activity for H_2_O_2_ decomposition	Generation of ROS and hyperthermal effects (PTT)	A metal organic framework (MOF) rich in porphyrin-like single atom Fe(III) centers (P-MOF) could efficiently induce cancer apoptosis under NIR irradiation and enabled photoacoustic imaging of cancer cells.
Wang et al. (2020) [26]	Single-atom Ru supported by Mn_3_[Co(CN)_6_]_2_ MOF	Mn_3_[Co(CN)_6_]_2_	Ru (2.23 wt% loading ratio)	In situ one-pot multicomponent self-assembly	XRD, HAADF-STEM, XPS, NEXAFS, TEM, DLS, UV-Vis	Biosafety (blood biochemical and blood routine)	Breast cancer (4T1 tumor -bearing mice)	4T1 tumor cell, HeLa cell, HEK 293 cell	Both	Reaction rate constant (0.041 min^−1^) higher than MnO_2_ (0.029 min^−1^)	Offered continuous O_2_ supply for hypoxia amelioration, enhanced efficacy of PDT, and ROS	OxgeMCC-r SAE with single-atom Ru loading content could relieve the hypoxia condition of solid tumors, lead to an enhanced ROS generation, and cause apoptotic cell death both in vitro and in vivo. OxgeMCC-r SAE could selectively accumulate within tumor sites for enhanced photodynamic therapy of cancer under the guide of T1 MR imaging.
Lu et al. (2020) [27]	Single-atom Cu coordinated in hollow N-doped carbon sphere	Hollow N-doped carbon sphere	Cu (0.18 wt.%)	Organic coating, carbonization, and silica etching	AC HAADF-STEM, HR-TEM, TEM, SEM, XRD, EDS, Selected area diffraction (SAED), ICP-OES, XANES, EXAFS, XPS, Raman, FTIR	High biocompatibility (body weight)	Breast cancer (4T1 tumor -bearing mice)	4T1 tumor cells	Both	Turnover frequency (TOF) is ≈5000 times higher than that of Fe atoms in commercial Fe_3_O_4_	Concurrent generation of two types of ROS	The Cu-HNCS demonstrated significant toxicity against cancer cells in vitro. In addition, Cu-HNCS catalysts could effectively suppressed tumor growth and significantly increased the survival rates in vivo.

**Table 2:** Application of SACs on anti-infection therapy.

Author	Type of SAC	Support Material	Active Site (Alloy Atom)	Synthesis Method	Characterization Techniques	Biocompatibility/Biosafety	Application (Model)	Targeted Bacteria	In Vitro/ In Vivo	Activity	Mechanism	Main Findings
Huang et al. (2019) [17]	Single-atom nanozymes with carbon nanoframe-confined FeN_5_	Carbon	Fe (Fe 1.2 wt.%; N 4.8 wt.%)	Pyrolysis	SEM, TEM, XRD, FTIR, XAFS, XANES, Fourier transform (FT)-EXAFS, HAADF-STEM	NCM460 cells, CCK-8	Infection wound (mice)	*E. coli (G^−^)* and *S. aureus (G^+^)*	Both	0.44 µM/s, 17 times higher than the FeN_4_ SA/CNF; 70 times greater than Pt/C	Generation of ROS	FeN_5_ SA/CNF exhibited significantly higher oxidase-like activity than that of square planar FeN_4_ catalyst. Further, FeN_5_ SA/CNF showed efficient bactericidal properties in vitro and wound disinfection in vivo.
Xu et al. (2019) [18]	Zinc-based single atom nanozyme	Carbon	Zn (3.12 wt.%)	Pyrolysis	TEM, DLS, EDS, HAADF-STEM, EELS, ICP-OES, EXAFS, XPS	Not reported	Infection wound (mice)	*P. aeruginosa (G^−^)*	Both	PMCS presents affinity for H_2_O_2_ (*K_M_* =40.16 nM), has high peroxidase-like activity	Generation of ROS	PMCS SAzyme present excellent perodidase-like activity and inhibited the growths of *P aeruginosa* by up to 99.87% in vitro, promoted wound healing in vivo.
Huo et al. (2019) [19]	Single iron atoms anchored in nitrogen-doped carbon (SAF NCs)	N-doped carbon	Fe (1.36 wt.%)	Encapsulated-pyrolysis strategy	SEM, TEM, ESR, SAED, EDS, XAFS, FT-EXAFS, HAADF-STEM	Satisfied biocompatibility	Infection wound (Balb/mice)	*E. coli (G^−^)* and *S. aureus (G^+^)*	Both	Peroxidase-like activity of SAF NCs is 76.4 folds higher than Fe_3_O_4_ NPs (25 μg mL^−1^)	Generation of ROS, and in combination of PTT	SAF NCs exhibited highly efficient in vitro antibacterial property (*S. aureus* and *E. coli*) and in vivo anti-infection performance, resulting in better wound healing.
Cao et al. (2020) [20]	Nitrogen-doped carbon-supported atomically dispersed Co-porphyrin centers (Co/PMCS)	N-doped carbon	Co (2.02 wt%)	The pyrolysis of Co-doped ZIF-8 (bimetallic MOF	TEM, HRTEM, HAADF-STEM, HAADFS, EDS, XRD, ICP-OES, XPS, EXAFS, FT-/WT-EXAFS	N/A	Sepsis and bacteremia (mouse)	*E. coli (G^-^)*	Both	(1) superoxide dismutase (SOD)-like activity: 16429 Umm^−1^; (2) catalase (CAT)-like activity: 820 Umm^−1^; (3) glutathione peroxide (GPx)-like activity: 3.9 Umm^−1^	Elimination of ROS	Co/PMCS were superior to the existing antioxidative nanozymes, which could efficiently eliminate O_2_^-^ and H_2_O_2_, and reduce proinflammatory cytokines level, suppressing their ability to intensify the sepsis cascade.

**Table 3:** Application of SACs for trauma therapy and cytoprotection.

Author	Type of SAC	Support	Active Site (Alloy Atom)	Synthesis Method	Characterization Techniques	Biocompatibility/ Biosafety	Application	In Vitro/ In Vivo	Activity	Mechanism	Main Findings
Yan et al. (2019) [25]	Single-atom Pt/CeO_2_	Ultrasmall CeO_2_ cluster	Pt (2 wt.%)	Deposition/precipitation	HAADF-STEM, DFT, XRD, XPS, Raman, ESR, UV-vis-NIR	Good	Brain Trauma	Both	POD-like activity higher than CeO_2_ and Pt clusters; CAT-like activity and SOD-like activity higher than CeO_2_ clusters	Multienzyme activities and the scavenging activities against RONS	The single-atom Pt/CeO_2_ present long-lasting catalytic activity. Additionally, it exhibited the nanozyme-based bandage could decrease indicators of oxidative stress and inflammation responses of neuron cells and improve impaired neurocognition.
Lu et al. (2019) [22]	Fe–N4 immobilized on a carbon substrate (Fe-N/C SACs)	Carbon substrate	Fe (3.8 wt.%)	Fe-N/C SACs prepared by mechanical means and pyrolysis	XRD, HAADF-STEM, UV-Vis absorption	Good	HeLa cells	In vitro	Fe–N/C SACs exhibit excellent POD-like, OXD-like, CAT-like, and GPx-like catalytic activity	Scavenge intracellular ROS, excess H_2_O_2_	Fe–N/C SACs could be used as enzyme mimicking properties, including peroxide (POD)-like, oxide (OXD)-like, CAT-like, and GPx-like activities. In addition, it could effectively scavenge intracellular ROS in HeLa cells.
Ma et al. (2018) [15]	Fe-SAs/NC	N-doped porous carbon	Fe (0.25 wt.%)	Pyrolysis	XANES, EXAFS,	minimal cytotoxicity	HeLa cells	In vitro	Excellent CAT-like activity: Fe-SAs/NC TOF=1809.34 min^−1^, high SOD-like activity	Elimination of ROS by catalyzing the decomposition of H_2_O_2_	Fe-SAs/NC catalysts containing bifunctional Fe-N_4_ active sites showed excellent CAT-like and SOD-like activities. Moreover, it could protect HeLa cells efficiently against oxidative stress by eliminating H_2_O_2_ and O_2_.

## 4. Discussion

This is the first systematic review attempting to collate all empirical evidence on the effect of SACs in biotherapy applications. All of the included studies indicated that SACs had been successfully introduced in biotherapy fields. The results of this systematic review suggested that various types of SACs were synthesized, which exhibited exceptional performance in antitumor, wound disinfection, anti-inflammation, antibacterial, trauma therapy, and oxidative-stress cytoprotection applications. In addition, the vast majority of the synthesized SACs (90%) exhibited satisfied biocompatibility. These results indicated that SACs could be a promising candidate for therapeutic applications. The characterization of SACs, along with their therapeutic effects, mechanisms, limitations, and prospects are discussed below.

### 4.1. Formatting of Mathematical Components

SACs have received a great deal of attention since they offer unique advantages in regard to their high activity and selectivity for various catalytic reactions. Isolated single atoms act as the centers of the catalytically active sites of SACs [28,29,30]. It was reported that single-atom dispersion is a very important factor that contributes to the great performance of SACs when used in a wide variety of practical applications [30,31]. Nevertheless, SACs could not be clearly characterized owing to the limits of instrument resolution in the last few years. Until recently, the rapid development of characterization techniques, including STM, HAADF-STEM, EXAFS, and XANES, have been applied to precisely characterize SACs [29,32]. In the present review, HAADF-STEM and/or STEM were used to identify and confirm the single atoms of the synthesized SACs in all included studies. In addition, EXAFS or XANES were applied to elucidate more detailed information regarding the binding mode, coordination environment, and oxidation states of the single atom. It is worth mentioning that any identified studies that lacked the identification of their synthesized SACs were not included in this review. For instance, a study investigating the effect of an Fe-N-C artificial enzyme in a cancer treatment was excluded because the authors only suggested that Fe was atomically dispersed in Fe-N-C without key characterization evidence [33].

### 4.2. Structures of the SACs

In the present review, we found that several types of SACs were developed for biomedical applications due to the structural similarity between natural enzymes and SACs. For these SACs, both noble metals (Pt, Ru, and Au) and non-noble metals (Fe, Co, Cu, and Zn) were used as single atoms. Among them, an Fe single-atom catalyst was the most commonly used single atom in the included studies. Iron is essential for most life on the planet, playing a crucial role in cellular metabolism and ROS production. Therefore, Fe-containing catalysts are generally regarded as one of most promising candidates for the production of abundant toxic •OH radicals.

Apart from the single atom, the support material, which serves as the ligands of the active metal centers, is another crucial factor for the catalytic performance of SACs. Furthermore, the support could stabilize the single-metal atoms and actively participate in catalytic reactions [25]. In the present review, several types of supports were synthesized, including, carbon-based materials, MOFs, Mn_3_[Co (CN_6_)_6_]_2_, and CeO_2_. It was noted that carbon-based materials were one of the most commonly used supports of synthesized SACs, similar to SACs in the field of energy, chemical, environmental, and industrial fields. In other words, SACs supported by carbon exhibited excellent promise in the catalysis field, which is mainly due to their specific features, including but not limited to their superb chemical and mechanical dependability, good electrical and thermal conductivity, variable structural and morphological combinations, tunable porosity and surface properties, high specific surface area, easy handling, and low production cost. 

Typically, the supports of SACs are divided into two categories: 2D and 3D supports. The overwhelming majority of the studies (10 out of 12) synthesized 2D-supported SACs, and the single-atom loading ratios varied from 0.25 to 3.8 wt.%, which were higher than the carbon sphere support (0.18%) but much less than the 3D carbon-dot support (15.3 wt.%) in one study. However, Zhang and coworkers, who first introduced SACs, reported that 2D supports provided a better nanoplatform than their 3D-structured counterparts in regard to strengthening the metal-support interactions via chemical modifications [32]. The interactions between single metals and supports determine the loading ratio of a single metal and the performance of catalysts, thereby playing a vital role in stabilizing single-metal atoms and increasing activity and selectivity for the development of high-loading of SACs. In our review, one study was related to the 3D-supported SACs. In addition, different supports could offer different anchoring sites to stabilize single-atom metals. Unfortunately, it is unreasonable to conclude whether 3D-supported SACs are better for the biotherapy applications. In future studies, SACs with high metal-loading ratios on appropriate supports with outstanding stability, activity, and selectivity need to be developed for biotherapy applications.

### 4.3. Biotherapeutic Effect of SACs

In line with the superb performance in the energy, chemical, environmental, and industrial fields, all of the included studies in the present review consistently indicated that the synthesized SACs had superior therapeutic effects on several medical diseases and conditions when compared with that of the control. SACs have been applied for treating cancer (liver and breast tumors), infection wounds, sepsis and bacteremia, and brain trauma. In addition, SACs have also been used for oxidative-stress cytoprotection. It was suggested that SACs have great potential to become the next generation approach for the treatment of various medical disorders. However, there is currently a very limited number of studies published on the biomedical applications of SACs. In other words, no more than four published studies employed SACs for treating each type of the aforementioned medical condition. This might be because biotherapy application is a recent application of SACs. Therefore, additional studies that target various medical disorders with methodological rigor are needed to confirm the excellent performance of SACs in medical applications.

### 4.4. Mechanism of Biotherapy Applications Biotherapeutic Effect of SACs

To guide the rational design of high-performance SACs, the underlying therapeutic mechanisms of SACs on wound disinfection, cancer treatment, brain trauma therapy, and oxidative stress cytoprotection have been comprehensively elucidated in all the included studies. In general, reactive oxygen species (ROS) are chemically reactive chemical species containing oxygen, such as superoxide (O_2_^−^), singlet oxygen (^1^O_2_), hydrogen peroxide (H_2_O_2_), nitric oxide (NO), and highly reactive hydroxyl and monoxide radicals (^•^OH, NO^•^), and peroxynitrite (ONOO^−^), and these ROS play an essential role in the therapeutic applications of SACs. It was suggested that ROS could react with large amounts of biomolecules, including lipids, proteins, RNA, and DNA. 

In regard to cancer treatments, the synthesized SACs, including PSAF NCs, Au/CDs, P-MOF, OxgeMCC-r SAE, and Cu-HNCS, displayed excellent oxidase-like and/or peroxidase-like activities in acidic environments. The H_2_O_2_ molecule was adsorbed on the single-metal active sites in the SACs, and then the activated H_2_O_2_ molecules (H_2_O_2_^*^) decomposed into 2OH^*^ via the single-metal catalysts, which decreased the reaction energy barrier of homolysis (showed a lower reaction energy barrier than that of heterolysis). Interestingly, the bioinspired Cu-HNCS could concurrently catalyze the decomposition of both O_2_ and H_2_O_2_ to O_2_^•−^ and ^•^OH, respectively, thus leading to a satisfactory efficacy of this cancer therapy. Similarly, the mechanism of synthesized SACs, such as FeN_5_ SA/CNF, PMCS SA, and SAF NCs for anti-infection, was to produce ROS during the catalytic reduction of oxygen, and these ROS were able to seriously impair the membrane integrity of bacteria to enhance antibacterial efficiency.

In contrast, the mechanism of single-atom Pt/CeO_2_ for the brain trauma treatment was to scavenge RONS, exhibiting multienzyme activities such as peroxidase (POD)-like, catalase (CAT)-like, and oxide (OXD)-like catalytic activities. Similarly, the mechanism of synthesized SACs, such as Fe-N/C SACs, and Fe-SAs/NC for oxidative-stress cytoprotection, was to eliminate ROS and excess H_2_O_2_. 

During these biotherapy applications, single-metal atoms commonly behaved as heterogeneous catalysts and influenced the direction and activity of chemical reactions, thereby serving as excellent functional biomimetic enzymes and regulating the generation or elimination of ROS in various microenvironments.

### 4.5. Limitations and Perspectives

Despite their exciting and encouraging beginning, the biotherapy applications of SAC is still in its infancy. Several issues must be addressed to obtain an in-depth understanding of single-atom catalysis and eventually achieve the rational design of SACs for specific therapeutic applications. In the future, precisely controlling the metal-loading ratio and modifying the active sites of SACs will minimize the immune defense and improve the in vivo/in vitro catalytic efficiency. In addition, the biocompatibility of SACs is still a major obstruction for their biological applications. Despite that 11 out of 12 included studies reported that SACs exhibited satisfactory biocompatibilities in the biotherapy applications, the mechanisms of the biocompatibilities were not investigated and discussed. Furthermore, long-term biocompatibility evaluation of the SACs in the biotherapy field has not been explored. Theoretically, SACs might lead to prolonged blood circulation time and undesirable immune responses since SACs have a relatively larger size and higher stability than biomolecules. Therefore, more efforts are needed to regulate the size of SACs for obtaining more promising biotherapy applications with outstanding biocompatibility in the future. To date, only Fe-, Zn-, Co-, Pt-, Au-, and Ru-based SACs have been verified in the biotherapy applications; interestingly, other noble metal-based SACs, such as Pd, Ag, have yet to be evaluated. The development of other supports, such as metallic oxides and polymers, should also be further investigated.

## 5. Conclusions

The biotherapy application of SACs is a prospective research frontier. The included studies indicated that the synthesized SACs were effective in anticancer therapy, anti-infection (antibacterial and anti-inflammation) therapy, brain trauma therapy, and oxidative-stress cytoprotection. Furthermore, it is anticipated that remarkable improvements can be made in designing highly efficient SACs for various biotherapy applications and to achieve the desired goal of facilitating the clinical translation of SACs.
